# Acute IL-6 exposure triggers canonical IL6Ra signaling in hiPSC microglia, but not neural progenitor cells

**DOI:** 10.1016/j.bbi.2023.02.007

**Published:** 2023-05

**Authors:** Amalie C.M. Couch, Shiden Solomon, Rodrigo R.R. Duarte, Alessia Marrocu, Yiqing Sun, Laura Sichlinger, Rugile Matuleviciute, Lucia Dutan Polit, Bjørn Hanger, Amelia Brown, Shahram Kordasti, Deepak P. Srivastava, Anthony C. Vernon

**Affiliations:** a Department of Basic and Clinical Neuroscience, Institute of Psychiatry, Psychology and Neuroscience, King’s College London, London, UK; b MRC Centre for Neurodevelopmental Disorders, King’s College London, London, UK; c Department of Social, Genetic & Developmental Psychiatry, Institute of Psychiatry, Psychology & Neuroscience, King’s College London, London, UK; d Department of Medicine, Weill Cornell Medical College, Cornell University, NY, USA; e Division of Immunology, Infection and Inflammatory Disease, King’s College London, London, UK; f Comprehensive Cancer Centre, School of Cancer and Pharmaceutical Sciences, Faculty of Life Sciences and Medicine, King's College London, London, UK

**Keywords:** IL-6, Neurodevelopmental disorders, Human induced-pluripotent stem cells, Microglia, Neural progenitor cells

## Abstract

**Background:**

Prenatal exposure to elevated interleukin (IL)-6 levels is associated with increased risk for psychiatric disorders with a putative neurodevelopmental origin, such as schizophrenia (SZ), autism spectrum condition (ASC) and bipolar disorder (BD). Although rodent models provide causal evidence for this association, we lack a detailed understanding of the cellular and molecular mechanisms in human model systems. To close this gap, we characterized the response of human induced pluripotent stem cell (hiPSC-)derived microglia-like cells (MGL) and neural progenitor cells (NPCs) to IL-6 in monoculture.

**Results:**

We observed that human forebrain NPCs did not respond to acute IL-6 exposure in monoculture at both protein and transcript levels due to the absence of *IL6R* expression and soluble (s)IL6Ra secretion. By contrast, acute IL-6 exposure resulted in STAT3 phosphorylation and increased *IL6, JMJD3* and *IL10* expression in MGL, confirming activation of canonical IL6Ra signaling. Bulk RNAseq identified 156 up-regulated genes (FDR < 0.05) in MGL following acute IL-6 exposure, including *IRF8, REL, HSPA1A/B* and *OXTR*, which significantly overlapped with an up-regulated gene set from human *post-mortem* brain tissue from individuals with schizophrenia. Acute IL-6 stimulation significantly increased MGL motility, consistent with gene ontology pathways highlighted from the RNAseq data and replicating rodent model indications that IRF8 regulates microglial motility. Finally, IL-6 induces MGLs to secrete CCL1, CXCL1, MIP-1α/β, IL-8, IL-13, IL-16, IL-18, MIF and Serpin-E1 after 3 h and 24 h.

**Conclusion:**

Our data provide evidence for cell specific effects of acute IL-6 exposure in a human model system, ultimately suggesting that microglia-NPC co-culture models are required to study how IL-6 influences human cortical neural progenitor cell development *in vitro*.

## Highlights

• Neural progenitor cells do not express required machinery for IL-6 cis-signaling but microglia do.
• Acute IL-6 induces cell specific responses from neural progenitor and microglia cells.
• Microglia IL-6 transcriptome overlaps with schizophrenia post-mortem genesets.
• Microglia-NPC co-culture models are required to investigate the IL-6 effect on neurodevelopment.

## 1 Introduction

Maternal immune activation (MIA) during pregnancy is associated with a generalized increased risk of the offspring developing psychopathology later in life, including schizophrenia (SZ), bipolar disorder (BD), depression and autism spectrum condition (ASC) (Estes and McAllister, 2016, Mondelli et al., 2017). MIA is a broad term that covers multiple risk sources, covering both infectious and non-infectious stimuli (Meyer, 2019). Human epidemiological studies suggest MIA acts to increase the risk for psychopathology equally during both prenatal and postnatal periods (Lydholm et al., 2019), findings given causal support by data from animal models of MIA (Meyer, 2019, Potter et al., 2023). The more an offspring is exposed to such scenarios, the higher the risk for psychopathology, suggesting an additive mechanism (Bayer et al., 1999, Couch et al., 2021, Meyer, 2019). Fundamentally however, the mechanisms by which pre- and postnatal exposure confer disease risk are likely to be explained by distinct cellular and/or molecular mechanisms. Focusing specifically on *prenatal* MIA exposure, both human and rodent studies suggest that, at least in part, the maternal peripheral cytokine profile mediates the increased risk for psychopathology in the offspring (Allswede et al., 2020, Careaga et al., 2017, Graham et al., 2018, Meyer, 2014, Mueller et al., 2021, Potter et al., 2023, Rasmussen et al., 2021, Rasmussen et al., 2019, Rudolph et al., 2018). Whilst data from human, rodent and *in vitro* models are not always consistent, there is accumulating evidence to suggest that interleukin (IL-)6 may act as a sensor, effector, and transducer of environmental risk factors on the prenatal brain. This view is consistent with evidence from genetic and blood biomarker studies that implicate IL-6 in the pathogenesis of multiple psychiatric disorders (Graham et al., 2018, Ozaki et al., 2020, Perry et al., 2021, Rasmussen et al., 2021, Rasmussen et al., 2019, Rudolph et al., 2018, Smith et al., 2007).

Specifically, birth cohort data show that higher levels of maternal serum steady state IL-6 concentrations correlate with larger right amygdala volume and stronger bilateral amygdala connectivity in the offspring, which influence both cognitive development and some externalizing behaviors in the offspring (Graham et al., 2018, Rasmussen et al., 2021, Rasmussen et al., 2019, Rudolph et al., 2018). In a mouse model based on maternal exposure to the viral mimetic Poly I:C, transcripts of *IL6* are shown to be consistently elevated in maternal liver, placenta, and primary fetal microglia (Ozaki et al., 2020). Furthermore, peripheral IL-6 levels remain elevated in adult mouse offspring which exhibit behavioral deficits relevant for SZ and ASC after MIA exposure compared to offspring who do not show any such deficits, despite exposure to MIA *in utero* (Mueller et al., 2021). Moreover, acute elevation of IL-6 by injection into pregnant mice or developing embryos enhances glutamatergic synapse development resulting in overall brain hyperconnectivity and behavioral deficits relevant for ASC in adult offspring (Mirabella et al., 2021). Finally, blocking IL-6R signaling either genetically or pharmacologically in the pregnant rodent dam, irrespective of the immune stimulation paradigm, eliminates the pathological effects of MIA in the fetal rodent brain and subsequent behavioral deficits in the adult animal (Smith et al., 2007).

Human epidemiological and neuroimaging studies cannot however establish the cellular or molecular basis underlying the effects of exposure to elevated levels of IL-6 prenatally. Whilst animal models address this gap, and have provided important causal mechanistic evidence, the extent to which data from such models may translate to humans remains unclear, due to the species-specific gene regulation networks that encompass human neurodevelopment (Yokoyama et al., 2014). This is compounded by heterogeneity between laboratories in the gestational timing, dose, frequency, and route of administration of the infectious challenge in rodents (Smolders et al., 2018) and batch-to-batch heterogeneity of infectious agents (Mueller et al., 2019). As such, conflicting findings exist in the animal MIA literature regarding cellular mechanisms (Kentner et al., 2019), exemplified by studies on the role of microglia (Smolders et al., 2018). Third, only a fraction of animal studies has investigated cellular or molecular phenotypes proximal to the MIA event in the developing brain. This is important as knowledge of the most proximal molecular events to MIA could reveal important therapeutic targets for prevention of downstream pathology.

Human induced pluripotent stem cells (hiPSC), which may be differentiated into multiple different neural and glial lineages, have the potential to address these gaps in our knowledge. Specifically, hiPSC directed towards neuronal fates have been utilized to investigate the pathological impact of Zika virus infection (Muffat et al., 2018), exposure to TLR3-agonists (Ritchie et al., 2018), and following direct exposure to cytokines, including interferon-gamma (Warre-Cornish et al., 2020) and IL-6 (Kathuria et al., 2022). Nonetheless, these studies have exclusively focused on neurons or astrocytes, at the expense of human microglia (Bhat et al., 2022, Park et al., 2020, Russo et al., 2018, Warre-Cornish et al., 2020). We therefore lack data on the impact of IL-6 on these critical immune-effector cells in a human–relevant model. Converging lines of evidence from human genetics, brain *post-mortem* tissue studies, neuroimaging and peripheral biomarker studies implicate microglia and the innate immune system in the pathophysiology of neurodevelopmental disorders (NDDs) (Coomey et al., 2020, Mondelli et al., 2017). Since microglia also play critical roles in shaping neurodevelopment and the central immune response to maintain homeostasis (Hanger et al., 2020, Paolicelli et al., 2011), incorporating human microglia into hiPSC models to study the effects of immune activation on development is vital (Gonzalez et al., 2017, Russo et al., 2018).

To this end, we evaluated whether, and how, hiPSC-derived microglia-like cells (MGLs) and neural progenitor cells (NPCs) respond to acute IL-6 stimulation in monocultures. We considered the following four questions: (1) do the cells have the receptor machinery to respond to IL-6 and other cytokines; (2) do these cells respond to acute IL-6; (3) does acute IL-6 induce a transcriptional profile similar to that seen in the major psychiatric disorders; and finally, (4) how does acute IL-6 impact the function of human MGLs?

## 2 Materials and methods

### 2.1 Cell culture

Participants were recruited and methods carried out in accordance with the ‘Patient iPSCs for Neurodevelopmental Disorders (PiNDs) study’ (REC No 13/LO/1218). Informed consent was obtained from all subjects for participation in the PiNDs study. Ethical approval for the PiNDs study was provided by the NHS Research Ethics Committee at the South London and Maudsley (SLaM) NHS R&D Office. HiPSCs were generated and characterized from a total of nine lines donated by three males with no history of neurodevelopmental or psychiatric disorders (Supplementary Table 1) as previously described (Adhya et al., 2021, Warre-Cornish et al., 2020) and grown in hypoxic conditions on Geltrex™ (Life Technologies; A1413302) coated 6-well NUNC™ plates in StemFlex medium (Gibco, A3349401) exchanged every 48 h. For passaging, cells were washed with HBSS (Invitrogen; 14170146) and then passaged by incubation with Versene (Lonza; BE17-711E), diluted in fresh StemFlex and plated onto fresh Geltrex-coated 6-well NUNC^TM^ plates. For specifics on cell culture differentiation, see supplementary. Both cell types were differentiated from hiPSCs using an embryonic MYB-independent method (Haenseler et al., 2017, Shi et al., 2012). Day 14 MGL monocultures for dose response experiments were exposed for 3 h to 100 ng/ml, 10 ng/ml, 1 ng/ml, 100 pg/ml, 10 pg/ml, 1 pg/ml, 0.1 pg/ml IL-6 (Gibco; PHC0066) or 100 pM acetic acid vehicle and were collected immediately for analysis. MGL progenitor (D1) and MGL (D14) cultures for single, high dose IL-6 stimulation received 100 ng/ml IL-6 (Gibco; PHC0066) or 100 pM acetic acid vehicle stimulation for either 3- or 24-hours and were immediately collected for analysis. NPC cultures received 100 pM acetic acid vehicle or 100 ng/ml IL-6 (Gibco; PHC0066) on day 18 for 3 h, then were collected for analysis. Eighteen days after neural induction reflects early second trimester neurodevelopment, which corresponds to a known period of increased risk for offspring NDD in mothers with increased IL-6 serum concentrations (Estes and McAllister, 2016).

### 2.2 RNA extraction, cDNA synthesis and quantitative PCR

Cells cultured for RNA extraction were collected in room temperature TRI Reagent™ Solution (Invitrogen; AM9738) and stored at −80 °C. RNA was extracted as directed per manufacturer’s instructions. Precipitation of RNA by 0.3 M Sodium-acetate and 100 % ethanol at −80 °C overnight was performed to clean samples further, before resuspension in RNAse-free water. Nucleic acid content was measured using NanoDrop™ One. Reverse transcription of RNA to complementary DNA was carried out according to manufacturer’s instruction (SuperScript^TM^ III Reverse Transcriptase Invitrogen 18080093 and 40 U RNaseOUT Invitrogen 10777019). qPCR was carried out using Forget-Me-Not™ EvaGreen® qPCR Master Mix (Biotium; 31041-1) in the QuantStudio 7 Flex Real-Time PCR System (Fisher), according to cycling parameters described in Supplementary Table 6. Cycle threshold (Ct) data were normalized to an average of *GADPH*, *RPL13* and *SDHA* housekeeper expression Ct values, which were unchanged upon IL-6 stimulation according to bulk RNAseq normalized DESeq2 counts (unpaired *t*-test *p* = 0.281, *p* = 0.718 and *p* = 0.653 respectively) (Supplementary Fig. 3). Gene expression fold change analysis was calculated following the 2^−^ΔΔ^Ct^ method (Livak and Schmittgen, 2001), using the following formulas:

$$\mathrm{Ct}={\mathrm{Ct}}_{\mathrm{Gene}\;of\;interest}-{\mathrm{Ct}}_{\mathrm{Housekeeping}\;gene\;average}$$

$$\Delta \Delta Ct={\Delta Ct}_{\mathrm{Sample}}-{\Delta Ct}_{\mathrm{Control}\;average}$$

$$\mathrm{Fold}\;change\;from\;control=2^{-\Delta \Delta Ct}$$

### 2.3 Western blot

Cells were scraped on ice and collected in RIPA buffer (Supplementary Table 7), sonicated at 40 % for 10 pulses, pelleted for 15 min at 4 °C and proteins collected in supernatant. Protein concentration was quantified using the Pierce™ BCA protein assay kit (Thermo-Fisher; 23227). In preparation for SDS-PAGE separation, protein samples were denatured in Laemmli buffer and boiled at 95 °C for 5 min. 2 µg of each protein sample was loaded into self-made 10 % gels, alongside 5 µl of the Dual Color (BioRad #1610374) standards marker. Gels were run at 20 mA for approximately 20 min, then increased to 100 V until the samples reached the bottom of the unit (∼90 min). Separated samples were transferred to a PVDF membrane and run overnight at 78 mA in 4 °C. Blots were blocked in 5 % BSA TBS-T for 1 h at RT with agitation. Antibodies were diluted in blocking buffer; primary antibody incubation occurred overnight at 4 °C with agitation, and secondary antibody incubation at RT for 1 h with agitation (Supplementary Table 4). Washes between antibody probes occurred in TBS-T at three 15 min intervals. For visualization, ECL Western Blotting Substrate (GE Healthcare; RPN2106) was incubated on the blot at RT for 5 min before image capture by the Bio-Rad Molecular Imager® Gel Doc™ XR System. Signals were quantified using ImageStudioLite (LI-COR, version 5.2.5), by defining an identical rectangular region of interest around each signal band and measuring the median signal value which was then backgrounded against the automatic background detection of the software. Signal data for pSTAT3 was divided by the tSTAT3 signal data within each lane, to give a pSTAT3/tSTAT3 ratio in both vehicle and treated samples across all four timepoints (15, 30, 60 and 180 min). Fold change was calculated by dividing pSTAT3/tSTAT3 of treated samples with the matching timepoint pSTAT3/tSTAT3 vehicle data from identical membranes, to reduce batch effects across donors on different membranes. For example, the 15 min IL-6 treated pSTAT3/tSTAT3 ratio was divided by the 15 min IL-6 vehicle pSTAT3/tSTAT3 ratio to get a fold change from vehicle within timepoint.

### 2.4 RNA Library preparation and NovaSeq sequencing

Total RNA extracted from 3 h IL-6 treated day 14 MGLs was pooled from two clones per healthy male donor (n = 3 in total). The samples were submitted for sequencing at Genewiz Inc (South Plainfield, NJ). Libraries were prepared using a polyA selection method using the NEBNext Ultra II RNA Library Prep Kit for Illumina following manufacturer’s instructions (NEB, Ipswich, MA, USA) and quantified using Qubit 4.0 Fluorometer (Life Technologies, Carlsbad, CA, USA). RNA integrity was checked with RNA Kit on Agilent 5300 Fragment Analyzer (Agilent Technologies, Palo Alto, CA, USA). The sequencing libraries were multiplexed and loaded on the flowcell on the Illumina NovaSeq 6000 instrument according to manufacturer’s instructions. The samples were sequenced using a 2x150 Pair-End (PE) configuration v1.5. Image analysis and base calling were conducted by the NovaSeq Control Software v1.7 on the NovaSeq instrument. We obtained an average of 23.5 million 289-base pair paired-end reads per sample (Supplementary Table 8). All downstream analyses were carried out in R version 4.0.2 (R Core Team, 2020). FASTQ files were quality controlled using Fastqc (Wingett and Andrews, 2018) and subsequently aligned to the human reference genome (GRCh38) with STAR (Dobin et al., 2013). A count table was prepared and filtered for counts ≥ 1 using featureCounts (Liao et al., 2014) from the Rsubread (Liao et al., 2019a) package, version 2.4.3. Differential gene expression analysis was carried out using DESeq2 (Love et al., 2014) version 1.30.1 and the default Wald test. Subsequently, using the Benjamini-Hochberg (BH) method, only genes with adjusted P < 0.05 were considered differentially expressed and submitted for downstream analyses.

### 2.5 Enrichment analyses

Gene ontology (GO) analysis was carried out using WebGestalt (Liao et al., 2019b), where differentially expressed genes were tested for over representation of non-redundant cellular component, biological process and molecular function GO terms. This analysis used as a background list all genes considered expressed in our model, according to DESeq2s’s internal filtering criteria (i.e., adjusted P ≠ NA). Enrichment P-values were corrected for multiple testing using the Benjamini-Hochberg (BH) method, and only terms with adjusted P < 0.05 were considered significant.

Outcomes from differential expression analysis were uploaded into the Qiagen Ingenuity Pathway Analysis (IPA) software (QIAGEN Inc., https://digitalinsights.qiagen.com/IPA) to identify canonical pathways. Analysis-ready genes were selected by p ≤ 0.05 and logfold changes −0.06 ≤ or ≥ 0.06, resulting in 153 up-regulated and 22 down-regulated genes. Core analysis was filtered by human data and removed any cancer cell lines as reference from the IPA knowledge base (IPKB). Top 10 enriched canonical pathways were filtered by z-score ≥ |2|, an IPA measure of pathway directionality, and ordered by p-value adjusted by Benjamini-Hochberg (BH) corrections.

To calculate the overlap significance between genes up- or down-regulated in our model with those up- or down-regulated in *post-mortem* brain samples from SZ, BD or ASC cases (Gandal et al., 2018), we performed Fisher’s exact tests using the R package ‘GeneOverlap’ (Shen, 2021). We considered the number of genes expressed in our model according to DEseq2′s internal filtering criteria as the genome size. We performed multiple testing correction using the false discovery rate (FDR) method and considered significant results under the FDR of 0.01 (FDR < 1 %). We used MAGMA 1.10 (de Leeuw et al., 2015) to test whether genes differentially regulated in the IL-6 model overlapped with genes enriched with GWAS-supported risk variants. Briefly, MAGMA calculates gene-level enrichment by generating a gene-wide statistic from summary statistics, adjusting associations for gene size, variant density, and linkage disequilibrium using the 1000 Genomes Phase 3 European reference panel. Summary statistics from three GWAS studies (Grove et al., 2019, Mullins et al., 2021, Trubetskoy et al., 2022) were downloaded from the Psychiatric Genomics Consortium website. We analyzed only biallelic single nucleotide polymorphisms with minor allele frequency > 5 % and imputation score > 0.80. We excluded from this analysis all genes and variants that were located within the extended MHC locus on chromosome 6, between 25 and 34 Mb (Watanabe et al., 2017). SNPs from GWASs were assigned to genes using an annotation window of 10 kb upstream and downstream of each gene, using the gene annotation provided by the authors.

### 2.6 Motility assay

The motility of MGLs from donors M3_CTR_36S, 127_CTM_01 and 014_CTM_02, averaged from three harvests was measured by live imaging, with 6 technical repeat wells per condition. D0 macrophage/microglia progenitors were seeded onto a glass bottom 96 well plate (PerkinElmer) precoated with Poly-d-Lysine (Gibco; A3890401) at 22,000 cells/well and matured in MGL media for 14 days. On the day of imaging cells were exposed to 3 conditions: unstimulated, 100 pM acetic acid vehicle or 100 ng/ml IL-6 for 3 h. A complete media change was performed with microglia media containing either 100 ng/ml IL-6 or 100 pM acetic acid vehicle. Thirty min before imaging, a second complete media change was performed on all wells with microglia media containing either 100 ng/ml IL-6, 100 pM acetic acid vehicle or neither (unstimulated) in order to stain all cells for 30 min with HCS NuclearMask™ Blue Stain (Invitrogen; H10325) and CellMask™ Orange Plasma membrane Stain (Invitrogen; C10045). Immediately before imaging, the media containing treatment and stain was removed and replaced with FluoroBrite™ DMEM (Gibco; A1896701) imaging media without phenol. Cells were imaged for 2 h on an Opera Phoenix high throughput imaging system (Perkin Elmer) using a 20× objective over 5 consistent fields of view per well, and data was analyzed using Harmony High-Content Image analysis software (PerkinElmer).

### 2.7 Media cytokine array

Day 14 MGL media samples collected after 3 and 24 h of IL-6 exposure and pooled from one donor over 3 harvests were incubated with Proteome Profiler Human Cytokine antibody array membranes (R&D Systems; ARY005B), as per the manufacturers instructions. Dot blot signals were quantified using the Protein Array Analyzer Palette plug-in for ImageJ, and technical dot replicates averaged to one value. These values were backgrounded and normalized to positive reference controls on the dot blot.

### 2.8 sIL6Ra ELISA

The IL-6 Receptor (Soluble) Human ELISA Kit (Invitrogen; BMS214) was used as per the manufactures instructions to quantify soluble IL6Ra expression in day 14 MGL and day 18 NPC vehicle/treated cell culture media. Optical density (OD) was blanked and measured at 450 nm.

### 2.9 Statistical analysis

To account for variability between cultures, three distinct male donors considered as biological replicates, averaged from three technical replicate clone cultures per donor unless stated otherwise (Supplementary Table 1). The use of multiple clones per line enhanced reproducibility and ensured validation of results in each donor line. All statistical analyses were performed in Prism 9 for macOS version 9.3.1 (GraphPad Software LLC, California, USA), apart from RNAseq analyses which were carried out using the research computing facility at King’s College London, *Rosalind*, and R version 4.0.2 (R Core Team, 2020). Each specific test carried out is described in each corresponding figure legend, as well as the number of replicates hiPSC lines that make up each technical and biological replicates. Statistical summary tables can be found in the Supplementary. When comparing means for more than 2 groups (Supplementary Tables 11, 14, 15 and 20), one-way ANOVA was used. To test whether transcript expression changed after treating cells with IL-6, we performed unpaired t-tests (Supplementary Tables 17 and 19). When comparing means for two separate conditions (Supplementary Tables 9, 10 and 18), two-way ANOVA was used. *Post-hoc* testing was carried out using Benjamini method 5 % or 1 % FDR. P- and adjusted P-values < 0.05 were considered as statistically significant. During GO analysis, 1 % FDR cut off was chosen to concentrate the number of significantly associated pathways. During MGL quality control (Supplementary Fig. 1), two-way ANOVAs comparing each gene expression with donor and time point demonstrated cell phenotype was not influenced by donor line (Supplementary Tables 9 and 10). Therefore, to reduce batch and reprogramming variability, biological replicates were considered as separate male donors which were averaged from N = 3 technical replicates from either clone cultures of the same donor or separate MGL harvests, as described in figure legends. Statistics were not applied to media cytokine array data since the sample power was too low.

## 3 Results

### 3.1 Human iPSC-derived microglial-like cells express IL-6 signaling receptors, but cortical neural progenitor cells do not

**Fig. 1:**
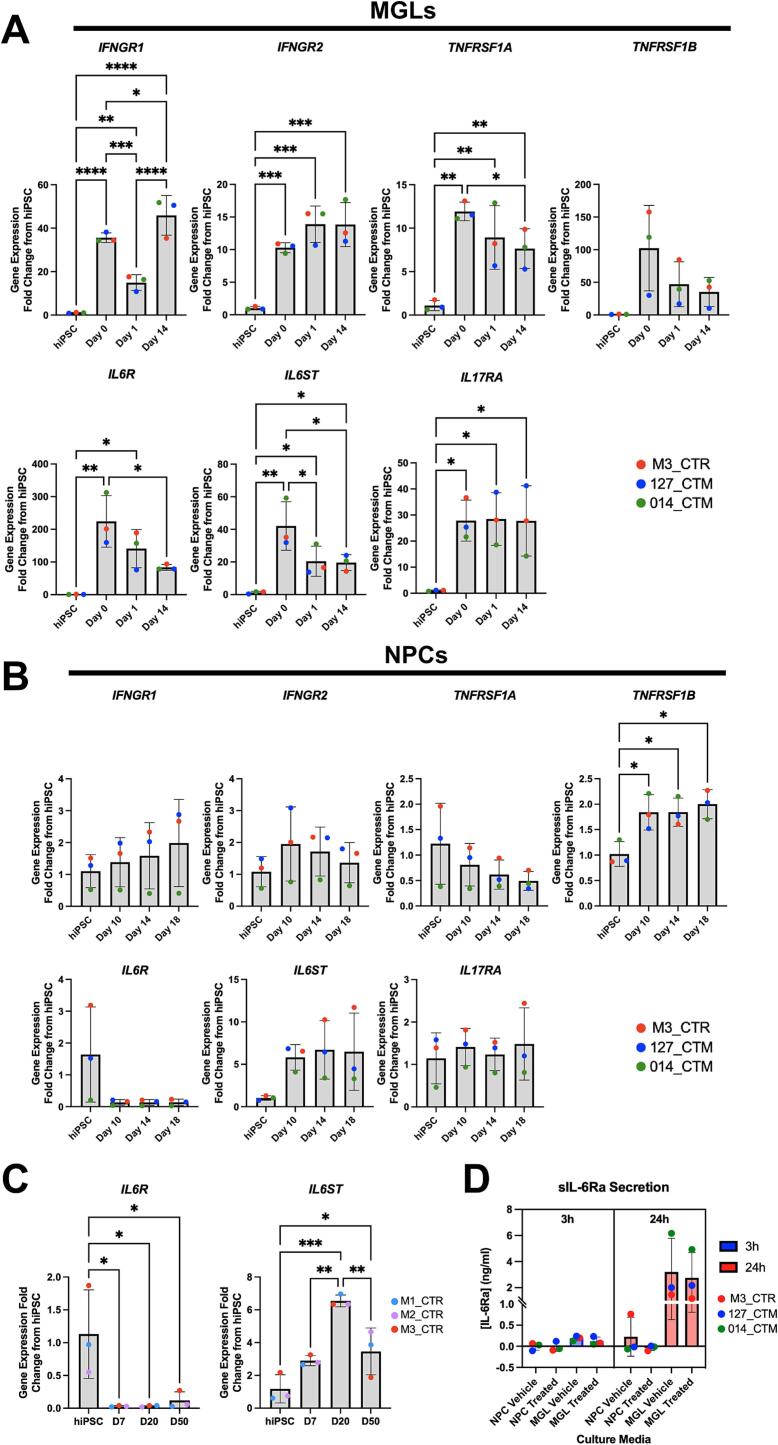
Cytokine receptor transcript expression in MGL and NPCs. Data shown are from N = 3 male neurotypical hiPSC cell lines, averaged from three technical replicate clones per donor, barring outlier removal where stated. 5 % false discovery rate (FDR) by Benjamini–Hochberg (BH) method corrections after one-way ANOVA formatted as follows: *p < 0.05, **p < 0.01, ***p < 0.001, and ****p < 0.0001; non-significant not labelled. Bar graphs plotted as mean with standard deviation (SD) error bars, and points colored by donor line as shown in key. (A) MGL differentiation time-course of cytokine receptors (*IFNGR1, IFNGR2, TNFRSF1A, TNFRSF1B, IL6a, IL6ST and IL17RA*) gene expression at hiPSC, macrophage/microglia progenitors (day 0), MGL at day 1 and day 14 of differentiation. M3_CTR day 14 *IL17RA* data point averaged from N = 2 clones only. (B) NPC differentiation time-course of the same cytokine receptors by qPCR RNA samples at hiPSC and days 10, 14 and 18 of neuralization to NPCs. 127_CTM day 14 *TNFRSF1B* data point was averaged form N = 2 clones only. (C) qPCR of *IL6R* and *IL6ST* transcript expression in N = 3 different healthy male lines (M1_CTR, M2_CTR and M3_CTR, one technical repeat each) over a longer timeframe, from hiPSC to D50 mature neurons. (D) Protein concentrations quantified by ELISA of soluble IL-6R (ng/ml) in NPC (on D18) and MGL (on D14) culture media after 3 h and 24 h vehicle or IL-6 100 ng/ml treatment exposure.

Successful differentiation of hiPSCs to MGLs and forebrain NPCs was confirmed by expression of key signature genes and proteins for each cell type (Supplementary Fig. 1). We then profiled human iPSC-derived MGL and NPC monocultures (N = 3 neurotypical male donors with N = 3 separate clones per donor) for cytokine receptor expression by qPCR to establish the potential of each cell type to respond to IL-6, and other cytokines, *in vitro* (ΔCt values available in Supplementary Tables 12 and 13). Transcript expression of *IFNGR1/2, TNFARSF1A, IL17RA*, and both subunits required for IL-6 signaling, *IL6R* and *IL6ST*, all significantly increased with longer differentiation of MGLs *in vitro* relative to the hiPSC state (Fig. 1A, statistics in Supplementary Table 14). Expression of *TNFRSF1B* was not significantly different from the hiPSC stage overall, but was numerically increased at all time points after MGL differentiation, namely ∼100-fold from hiPSC to D0, then ∼50-fold from hiPSC to D1 and ∼35-fold hiPSC to D14 (Fig. 1A). These data indicate that MGLs would be responsive to at least IL-6, IFNγ, TNFα and IL-17.

In contrast to MGLs, differentiation of hiPSCs to forebrain NPCs in monoculture led to extremely low levels of *IL6R* expression, reduced by ∼16-fold relative to the hiPSC stage, although this failed to reach statistical significance overall (Fig. 1B statistics in Supplementary Table 15). This observed reduction of *IL6R* expression upon differentiation is in agreement with previously reported data from human *post-mortem* fetal brain tissue (Fietz et al., 2012, Florio et al., 2015, Zhang et al., 2016) (Supplementary Fig. 2). Specifically, these data demonstrate *IL6R* is primarily expressed by microglia and in part by astrocytes in the human brain, but not in neurons (Miller et al., 2014, Zhang et al., 2016) (Supplementary Table 16). Transcripts for *IFNGR1/2, IL6ST* and *IL17RA* were expressed in forebrain NPCs, with expression levels either remaining constant or trended to increase (*p* > 0.05) throughout neuralization (Fig. 1B). *TNFRF1A* trended to decrease from hiPSC (*p* > 0.05) and *TNFRSF1B* increased significantly (*p* = 0.0127) relative to the hiPSC state (Fig. 1B, statistics in Supplementary Table 15). For a cell to respond to IL-6, the IL6Ra must be present in its membrane bound form along with IL-6ST (*cis*-IL6Ra signaling), or the soluble (s)IL6Ra form (*trans-*IL6Ra signaling), which is cleaved at the membrane surface of expressing cells is required (Wolf et al., 2014). These data indicate forebrain NPCs are likely to be unresponsive to IL-6 via *cis*-IL6Ra signaling when grown in monoculture, but responsive to IFNγ, as shown previously (Bhat et al., 2022, Warre-Cornish et al., 2020).

Contrary to this view, a recent study provides data to suggest that transcriptional and morphological phenotypes may be induced in hiPSC-derived D60 mature cortical pyramidal neurons following exposure to IL-6 at D25-27 of differentiation, using an identical protocol (Kathuria et al., 2022). We therefore sought to replicate our finding of very low *IL6R* expression and extend this analysis to longer differentiation times using an identical dual SMAD inhibition protocol, with N = 3 different hiPSC lines from male, neurotypical donors. Analysis of RNA samples by qPCR confirmed the very low expression of *IL6R* in forebrain NPCs and provides evidence to suggest this continues to be the case in mature neurons, at least at 50-days of differentiation *in vitro* (one-way ANOVA: F(3,8) = 7.30, *p* = 0.0112; Mean fold change from hiPSC: D7 = 0.03, D20 = 0.03, D50 = 0.12; Fig. 1C). By contrast, *IL6ST* expression increased throughout all stages of differentiation (one-way ANOVA: F(3,8) = 19.87, *p* = 0.0005; Mean fold change from hiPSC: D7 = 2.90, D20 = 6.54, D50 = 3.46; Fig. 1C). These data confirm our previous observations of very low *IL6R* expression in forebrain NPCs and extend these to mature cortical neurons (D50).

Given the apparent very low level of *IL6R* transcript expression in forebrain NPCs under the conditions tested, we next examined the secretion of the soluble IL6Ra protein in both forebrain NPCs and MGLs after a 3 h exposure to IL-6, indicative of the ability to initiate *trans*-IL-6 signaling (Fig. 1D) (Campbell et al., 2014, Michalopoulou et al., 2004, Wolf et al., 2014). Using a sIL6Ra-ELISA kit in vehicle-treated cultures, we observed very limited secretion of sIL6Ra protein into the culture supernatant by forebrain NPCs (Fig. 1D). By contrast, sIL6Ra secretion was clearly present in vehicle-treated MGLs in the culture supernatant after both 3 h and 24 h (Fig. 1D). Both 3 h and 24 h exposure to IL-6 (100 ng/ml) did not increase the secretion of sIL6Ra protein in either cell type (two-way ANOVA 3 h: cell type F(1,8) = 9.35 *p* = 0.0156, treatment F(1,8) = 0.497 *p* = 0.5009, interaction F(1,8) = 0.350 *p* = 0.5709; 24 h: cell type F(1,8) = 9.39 *p* = 0.0155, treatment F(1,8) = 0.147 *p* = 0.7116, interaction F(1,8) = 0.0106 *p* = 0.9207). Nonetheless, the fact that MGLs secrete sIL6Ra provides an opportunity for other cell types within their vicinity to respond to IL-6 via soluble, non-membrane bound (*trans*-)IL6Ra signaling. By contrast, the lack of sIL6Ra secretion from NPCs confirms the fact that they do not express the soluble form of the IL-6 receptor and strongly suggests forebrain NPCs are unlikely to be responsive to IL-6 in monoculture under the conditions tested.

### 3.2 IL-6 activates the canonical STAT3 pathway in microglia-like cells but not forebrain neural progenitor cells

**Fig. 2:**
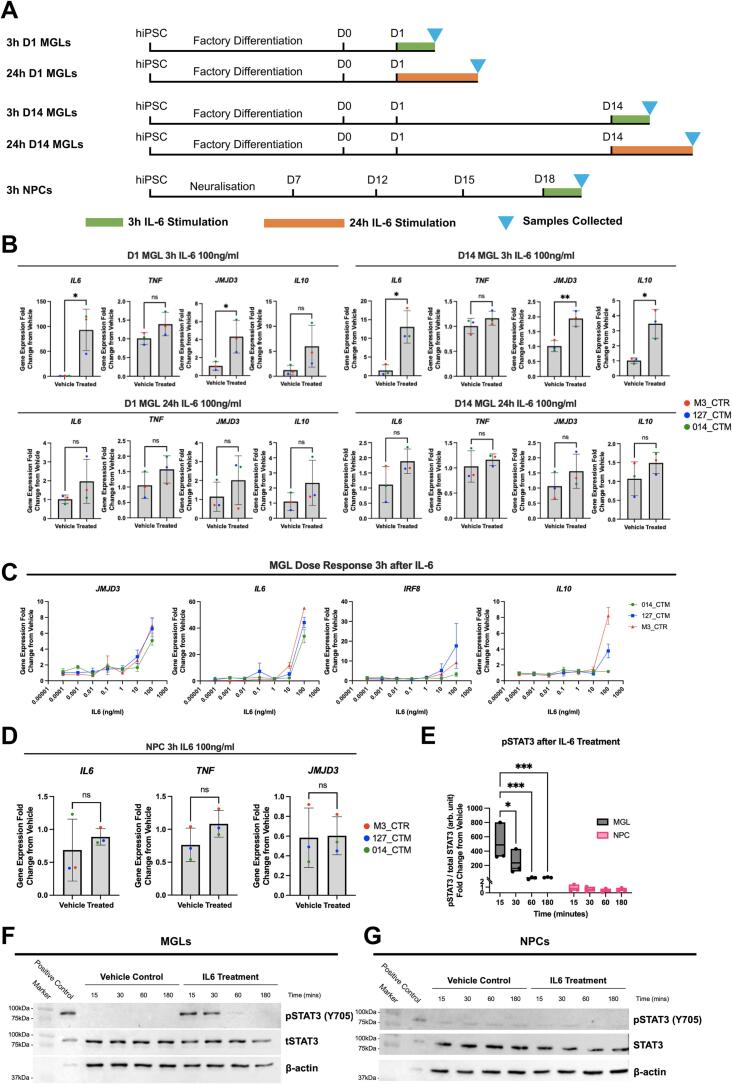
MGL monocultures respond to IL-6 in a dose and time dependent manner, NPC monocultures do not respond at all. (A) Schematic of MGL and NPC cell culture and RNA sample collection. (B) Transcripts of downstream IL-6 pathway genes *IL6, TNF, JMJD3* and *IL10* were measured by qPCR in three male healthy control cell lines treated with 100 ng/ml IL-6, averaged over three technical replicate clone cultures per donor unless stated otherwise, in the following conditions: D1 MGL treated for 3 h; D14 MGLs treated for 3 h; D1 MGL treated for 24 h, 014_CTM treated condition averaged from N = 2 clones only; MGLs treated for 24 h. Unpaired test results formatted as follows: *p < 0.05, **p < 0.01, ***p < 0.001, and ****p < 0.0001; not significant (ns). Bar graphs plotted as means with standard deviation (SD) error bars, and points coloured by donor line: red (M3_CTR), blue (127_CTM) and green (014_CTM). (C) Dose response of MGLs to 7 doses of a 10-fold serial dilution of IL-6 from 100 ng/ml to 0.1 pg/ml. Three healthy male donors with n = 3 harvest replicates per donor. *IL10* 127_CTM_01 0.1 ng/ml outlier removed and calculated form N = 2 harvests. Fold change from vehicle calculated within line, but vehicle not plotted. (D) Transcripts of downstream IL-6 pathway genes *IL6, TNF* and *JMJD3* in NPCs treated for 3 h, with unpaired *t*-test results formatted as above. *IL10* transcripts were undetectable in NPC samples so data is not shown. (E) Quantification of pSTAT3/tSTAT3 protein signal in arbitrary (arb.) units from blots F and G, shown as a fold change ratio from timepoint vehicle within cell type. Box plot presented with split y-axis at 2 arb. units to visualize variance in NPC data. (F-G) Immunoblotting for 88 kDa pSTAT3/tSTAT3 in both vehicle and 100 ng/ml IL-6 stimulated samples collected after 15, 30, 60 and 180mins, in MGL (F) and NPC (G) monocultures, with 3 h IL-6 100 ng/ml treated immortalized mouse microglia cells (BV2s) as a positive control.

We next determined the functionality of the IL6Ra and IL6ST receptors in forebrain NPCs and in MGLs at day 1 (D1) and day 14 (D14) of differentiation (Fig. 2A). First, transcripts of relevant STAT3 downstream target genes *IL6, IL10,* tumor necrosis factor α (*TNF)* and Jumonji Domain-Containing Protein 3 *(JMJD3)* (Przanowski et al., 2014) were measured by qPCR (Fig. 2B, statistics in Supplementary Table 17). At 3 h after exposure to IL-6 (100 ng/ml), both D1 MGL and D14 MGL cells responded by increasing the expression of *IL6* itself and *JMJD3*, relative to the vehicle control. Our modest sample size (N = 3 donors) may have reduced our ability, however, to detect any statistical significance when comparing *IL10* expression after IL-6 stimulation. For example, D1 MGL stimulated with IL-6 for 3 hr increase *IL10* expression (∼5-fold) more so than MGLs at the same time-point (∼3-fold), but the former result is not statistically significant whilst the latter is. The expression of *TNF* was not affected in either D1 MGL or D14 MGLs by IL-6 exposure (Fig. 2B, statistics in Supplementary Table 17). By contrast, at 24 h after exposure to IL-6 (100 ng/ml), the expression of all these genes was no longer statistically significantly different relative to the vehicle control in both D1 MGL and D14 MGLs (Fig. 2B). Based on the apparent functional maturity of MGLs after 14 days of differentiation (Haenseler et al., 2017), and their response to IL-6 by significantly increasing *IL10*, only MGLs differentiated for 14 days were used in all subsequent experiments.

Having confirmed IL-6 triggers a transcriptional response associated with IL6R signaling in MGLs, we next sought to determine the minimal concentration of IL-6 that would induce this response from day 14 MGLs in monoculture (Fig. 2C). We exposed day 14 MGLs to several different IL-6 concentrations (range: 0.1 pg/ml to 100 ng/ml) and measured the expression of genes that were up-regulated after 3 h (*IL6, IL10* and *JMJD3*) by qPCR. Our concentration range included the average, steady state concentration of IL-6 present in maternal serum collected from second-trimester mothers in a recent birth cohort study, which was reported to be 0.98 ± 1.06 pg/ml (Graham et al., 2018). We also measured expression of interferon regulatory factor 8 (*IFR8)*, a transcription factor known to regulate immune function and myeloid cell development (d’Errico et al., 2021, Tamura and Ozato, 2002). Expression of *IL6*, *JMJD3*, *IL10* and *IRF8* were unaffected relative to vehicle control at all concentrations of IL-6 tested except 100 ng/ml, which elicited a clear increase in expression, which varied between donors as may be expected (Fig. 2C, statistics in Supplementary Table 18). This 100 ng/ml IL-6 concentration is much higher than observed in steady state maternal serum (Graham et al., 2018) yet it would be expected that IL-6 concentration increases in maternal serum during an acute response to infection (Hsiao and Patterson, 2011, Matelski et al., 2021, Ozaki et al., 2020, Wu et al., 2017). For instance, adult patients with community-acquired pneumonia have a mean serum IL-6 of 477 pg/ml (Antunes et al., 2002), and COVID-19 patients with delirium have a mean serum IL-6 of 229.9 pg/ml (Borsini et al., 2022). We therefore selected 100 ng/ml IL-6 for further experiments since this elicited a response in our MGLs that can be measured at a single time-point, allowing us to investigate the response to IL-6, a specific cytokine that plays a key role in MIA, as per our previous work on IFNγ (Bhat et al., 2022, Warre-Cornish et al., 2020). Whilst this concentration is still significantly higher than the data reported above, it should be remembered that both microglia (consistent with our own data herein) but also astrocytes in the fetal brain would produce IL-6 in response to infection, thus local levels of IL-6 may be considerably higher than those reported in the maternal serum.

Since forebrain NPCs, in contrast to MGLs, displayed a very low level of *IL6R* expression, we sought to confirm whether forebrain NPCs in monoculture show any response to 100 ng/ml IL-6. Three hours after IL-6 exposure, forebrain NPCs did not significantly increase the expression of *IL6*, *JMJD3* and *TNF* transcripts relative to vehicle controls (Fig. 2D, statistics in Supplementary Table 19). Furthermore, the *IL10* transcript was undetectable in all NPC samples irrespective of treatment, given no CT values were generated (data not shown). These data suggest that whilst IL-6 triggers IL6R signaling in MGLs, this is not the case for forebrain NPCs at D18 *in vitro*.

To complement our gene expression analysis, we next assessed the time-scale of canonical STAT3 signaling following IL-6 receptor stimulation at the protein level in both forebrain NPCs and MGLs. Formation of the IL-6/IL6Ra/IL6ST complex on the cell surface membrane results in phosphorylation of STAT3 (pSTAT3) by the protein kinase JAK, which shuttles to the nucleus to enable subsequent transcription of STAT3 target genes (Wolf et al., 2014). We therefore collected protein samples at multiple time points following acute IL-6 exposure (100 ng/ml) of either NPCs or MGLs and performed western blotting for Y705-pSTAT3 and total STAT3 (Fig. 2E-G). Quantification of the Y705-pSTAT3 ratio to total STAT3 (tSTAT3) indicated that IL-6 triggered a time-dependent increase in pSTAT3 relative to vehicle controls that peaked after 15 min in MGLs but was absent in D18 forebrain NPCs (Fig. 2E, two-way ANOVA: cell type F(3,16) = 17.45; p = 0.0007, F(3,16) = 5.486; time p = 0.0089, interaction F(3,16) = 5.47; p = 0.0088).

### 3.3 Transcriptional response in human microglia-like cells to acute IL-6 exposure

**Fig. 3:**
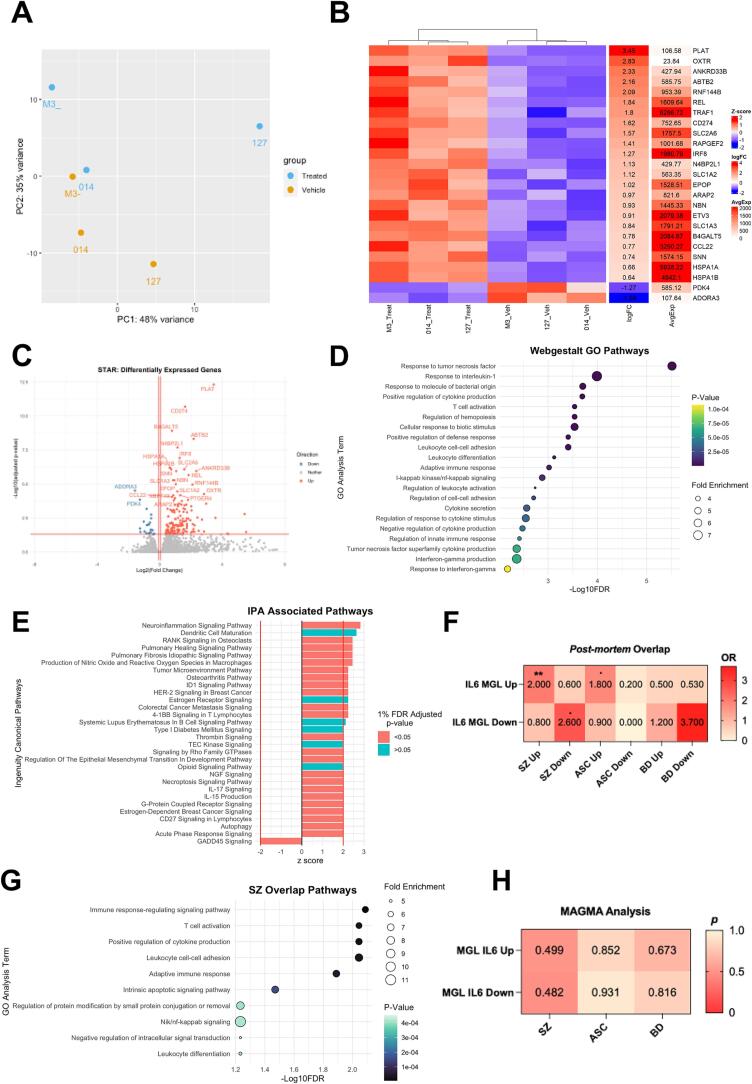
Acute IL-6 exposure elicits a transcriptional response in human microglia-like cells of relevance for schizophrenia. MGLs from 3 healthy male donors, pooled from 2 clone cultures each, were exposed to IL-6 or vehicle for 3 h and collected for RNAseq. (A) PCA analysis of all 6 samples, colored by vehicle (orange) or IL-6 treated (blue) condition and labelled by donor line: M3_CTR as M3-, 014_CTM as 014 and 127_CTM as 127. (B) Heatmap of top 25 most differentially expressed genes in the IL-6 3 h MGL response, ranked by LogFC and clustering by treatment group. (C) Volcano plot of differentially expressed genes. Conditional axis set as follows: log2Foldchange > 0.06 and adjusted p-value < 0.05 colored red; log2Foldchange < −0.06 and adjusted p-value < 0.05 colored blue. The top 25 differentially expressed genes are labelled. (D) Webgestalt gene ontology analysis of up-regulated 156 gene set only with an adjusted 1 % FDR. GO terms ordered by –log10FDR, colored by adjusted p-value and sized by the fold enrichment within each dataset. (E) IPA associated pathways, ranked by z-score and colored by 1 % FDR adjusted p-value. Only pathways with z-score > |2| are shown, with z-score > |2| conditional axes labelled in red. (F) Fisher’s exact test comparing gene sets from ASC,SZ and BD post-mortem human patient tissue (Gandal et al., 2018) with up- and down-regulated gene sets identified by RNAseq in this study. Odds ratios (OR) plotted in heatmap with significant 5 % FDR corrections formatted as follows: p < 0.1, *p < 0.05, **p < 0.01, ***p < 0.001, and ****p < 0.0001; not significant, not labelled. (G) Webgestalt gene ontology analysis of 44 genes overlapped from our up-regulated RNAseq dataset and SZ up-regulated genes from Gandal et al., 2018, with an adjusted 1 % FDR. GO terms ordered by −log10FDR, colored by adjusted p-value and sized by the fold enrichment within each dataset. (H) MAGMA analysis comparing significant risk genes from SZ (Trubetskoy et al., 2022), BD (Mullins et al., 2021) and ASD (Grove et al., 2019) GWAS studies with IL-6 MGL up and down DEGs obtained by RNAseq in this study. P-values plotted in heatmap.

Our data thus far provide evidence suggesting that the canonical STAT-3 signaling pathway is activated in MGLs within 3hr of exposure to IL-6. To better characterize the transcriptional response of MGLs to this stimulus, we next performed bulk RNA-sequencing at 3hr post IL-6 exposure (100 ng/ml) in MGLs generated from N = 3 male neurotypical donor hiPSC lines. Principal component analysis (PCA) of the gene expression data reveals that samples clustered by treatment (Fig. 3A), consistent with the heatmap clustering of the top 25 differentially expressed genes (DEGs) (Fig. 3B). Overall, we found that 156 and 22 genes were up- and down-regulated, respectively, following 3 h IL-6 exposure (FDR < 0.05) (Fig. 3B-C). Up-regulated genes of note included *IRF8,* consistent with our qPCR data (Fig. 2C) the NFkB subunit *REL,* heat shock proteins *HSPA1A/B* and the oxytocin receptor (*OXTR)* (Supplementary Fig. 3). Although maternal IL-17 is suggested to be involved in MIA-induced behavioral changes in offspring with relevance to ASC (Choi et al., 2016), we found no evidence for its differential expression in our MGL 3 h IL-6 response. The DESeq2 normalized counts (calculated using the median of ratios method) for *IL6R* and *IL6ST* remained constant, confirming that the expression of these receptors is independent of the 3 h IL-6 exposure (Supplementary Fig. 3). Using DESeq2 normalized counts we identified numerical increases in key IL-6 signaling transcripts, including STAT3, JAK1, JAK2, and JAK3, but not in tyrosine kinase 2 (TYK2), although none of these were statistically significant in the DESeq2 analysis (p > 0.05; BH 5 % FDR). Finally, the presence of microglial markers was confirmed in DESeq2 normalized counts, and no effects of IL-6 were observed on these markers (Supplementary Fig. 3).

Using only the 178 DEGs at 5 % FDR, we carried out Webgestalt GO analyses splitting these into either up-regulated (156) or down-regulated genes (22). Across cellular components, biological processes, and molecular functions, 21 GO pathways were significantly associated with the 156-up-regulated genes (1 % FDR) (Fig. 3D). These included the NFkB signaling (FE = 4.60, *p* < 0.001, FDR = 0.001), leukocyte differentiation (FE = 3.55, *p* < 0.001,FDR < 0.001), cell–cell adhesion regulation (FE = 3.77, *p* < 0.001, FDR = 0.002), response to cytokine stimuli (FE = 5.57, *p* < 0.001, FDR = 0.002), production of IFNγ (FE = 6.95, *p* < 0.001, FDR = 0.004) and TNF superfamily (FE = 5.87, *p* < 0.001, FDR = 0.004) cytokines (Fig. 3D). By contrast, no GO pathways at either 5 % or 1 % FDR correction were significantly associated with the 22 down-regulated genes. Complementary GO analysis using the QIAGEN Ingenuity Pathway Analysis (IPA) software (Krämer et al., 2014) identified 30 associated pathways at a z-score threshold of > 2 to identify predicted activation or inhibition of a pathway, of which 24 passed 1 % FDR correction (Fig. 3E). The top activated pathways were “neuroinflammation signaling” (Ratio = 0.035, *p* < 0.001, FDR < 0.001), nitric oxide and reactive oxygen species (ROS) in macrophages (Ratio = 0.037, *p* < 0.001, FDR < 0.001), TNFRSF signaling in lymphocytes (4-1BB: Ratio = 0.176, *p* < 0.001, FDR < 0.001; CD27: Ratio = 0.088, *p* < 0.001, FDR < 0.001), epithelial-mesenchymal transition in development (Ratio = 0.058, *p* < 0.001, FDR = 0.001), G-protein coupled receptor signaling (Ratio = 0.023, *p* < 0.001, FDR < 0.001), IL-17 signaling (Ratio = 0.021, *p* < 0.001, FDR = 0.034) and a down-regulation of GADD45 signaling (Ratio = 0.067, *p* < 0.001, FDR = 0.002). Overall, these complementary GO analyses provide evidence for a prototypical microglial cell response after 3 h of IL-6 stimulation, characterized by NFkB pathway activation and downstream pathway changes to ROS, cell adhesion, cytokine secretion and TNFRSF signaling.

### 3.4 The transcriptomic changes associated with IL-6 MGL exposure are associated with those observed in post-mortem brain tissue from schizophrenia cases

IL-6 is increased in human serum, cerebrospinal fluid (CSF) and *post-mortem* brain tissue in multiple psychiatric disorders including SZ, ASC, BD and major depressive disorder (MDD) (Gandal et al., 2018, Khandaker et al., 2014, Lu et al., 2019, Perry et al., 2021, Sasayama et al., 2013, Schwieler et al., 2015, Zhao et al., 2021). Furthermore, this association is demonstrated at multiple stages through life, including maternal serum (Allswede et al., 2020), in children aged 9 (Khandaker et al., 2014) and young adulthood at ages 18–25 (Schwieler et al., 2015). This is consistent with a view that IL-6 pathways are likely to be involved at different stages of the pathogenesis of psychiatric disorders. To corroborate the role of IL-6 signalling in these disorders, we therefore investigated whether the genes up- and down-regulated in MGLs following 3 h IL-6 stimulation were enriched within genes up- and down-regulated in *post-mortem* brain tissue from SZ, ASC and BD patients, as identified by Gandal et al. (2018) (two gene sets from the model vs two gene sets from each disorder: 12 comparisons in total; Fig. 3F). We observed statistically significant enrichments between the up-regulated gene set in the IL-6 model and the gene sets up-regulated in SZ (N genes in model = 156, N genes in cases = 2274, overlap size = 44 genes, P = 0.00022, odds ratio (OR) = 2.0) and ASC (N genes in model = 156, N genes in cases = 701, overlap size = 14 genes, P = 0.031, OR = 1.8), and between the down-regulated gene set in the model and the gene set down-regulated in SZ (N genes in model = 22, N genes in cases = 2073, overlap size = 7 genes, P = 0.04, OR = 2.6). The only significant overlap observed after multiple testing correction (1 % FDR), was that between the up-regulated gene set in the IL-6 model and the up-regulated genes in SZ cases (FDR = 0.003). Using the 44 overlapping genes, Webgestalt GO analyses identified 10 associated pathways (Fig. 3G, overlap gene list in Supplementary Table 20). This included signaling response pathways (FE = 6.61, *p* < 0.001, FDR = 0.008), “leukocyte cell–cell adhesion” (FE = 7.81, *p* < 0.001, FDR = 0.009), “*T*-cell activation” (FE = 6.40, *p* < 0.001, FDR = 0.009) and apoptotic signaling (FE = 7.29, *p* < 0.001, FDR = 0.034). Importantly, although just failing to pass a 5 % FDR correction, increased NFkB signaling was also highlighted as an output (FE = 11.4, *p* < 0.001, FDR = 0.059), consistent with gene expression data in post-mortem brain tissue from SZ patients as compared to controls (Volk et al., 2019). These data suggest IL-6 signaling modifies gene expression in a manner consistent with the dysregulation observed in the brains of individuals with SZ.

To complement this analysis, we next tested whether the genes differentially regulated in MGLs after 3 h IL-6 stimulation were significantly enriched in genes identified as risk factors for SZ, BD, and ASC through genome-wide association (GWAS) studies (Grove et al., 2019, Mullins et al., 2021, Trubetskoy et al., 2022). To this end, we performed gene-level enrichment analyses to identify risk genes associated with each condition, and we tested these for enrichment with the up- and down-regulated gene sets observed in the IL-6 stimulated MGLs, using MAGMA (de Leeuw et al., 2015). We found no significant enrichment for SZ (or any disorder) considering the genes differentially regulated in our model (all comparisons P > 0.05) (Fig. 3H). These data suggest that exposure of human MGLs to IL-6 does not alter the expression of genes associated with increased risk for SZ, BD and ASC.

Finally, we investigated the overlap of up- and down-regulated gene sets with microglia-specific module gene sets using the MGEnrichment tool (https://ciernialab.shinyapps.io/MGEnrichmentApp/) to compare our data with published gene expression data in human and mouse microglia (Jao and Ciernia, 2021). When comparing our data to microglia gene sets derived from human tissue only, we found 31 modules were associated with the up-regulated gene set and 12 with the down-regulated gene set after 5 % FDR correction from MGLs exposed to IL-6 for 3 h. Notable modules that overlapped with our up and down-regulated gene sets separately were a “SCZ, ASD and Bipolar Disorder (BD) module” (Up: intersection size = 44 genes, FDR = 7.10e-29, OR = 13.5. Down: intersection size = 8 genes, FDR = 5.69e-06, OR = 17.9) (Gandal et al., 2018), and a “core human microglial signatures module” (Up: intersection size = 26 genes, P = 3.29e-12, OR = 6.3. Down: intersection size = 10 genes, FDR = 4.47e-08, OR = 24.8) (Galatro et al., 2017), both of which originate from bulk RNAseq datasets. With the same tool, we additionally found both our up- and down-regulated gene set to be enriched for single-cell RNAseq microglial modules (Olah et al., 2020). These included up-regulation enrichment for “Microglia anti-inflammatory responses” (intersection size = 18 genes, FDR < 0.0001, OR = 9.90), “Microglia cellular stress” (intersection size = 7 genes, FDR < 0.0001, OR = 11.7), “Microglia interferon response signaling pathway” (intersection size = 8 genes, FDR = 0.0007, OR = 5.39) and “Microglia homeostatic states” (intersection size = 7 genes, FDR = 0.009, OR = 3.96). Our down-regulated gene set was enriched for “Microglia antigen presentation” (intersection size = 4 genes, FDR = 0.0003, OR = 29.2) and additionally “Microglia homeostatic states” (intersection size = 3 genes, FDR = 0.018, OR = 12.6). To assess the additional overlap with mouse data, we used the MGEnrichment tool and found 26 mouse modules to overlap with our up-regulated gene set and 4 mouse modules with our down-regulated gene set. The most relevant module that overlapped with our up-regulated gene set was “MIA Poly I:C GD14 P0” module (intersection size = 11 genes, FDR = 0.016, OR = 2.95) from Matcovitch-Natan et al. (2016), a microglial gene set taken from newborn pups whose mothers were exposed to Poly I:C on gestational day 14 (Matcovitch-Natan et al., 2016). Of note, the intersection size of this comparison is only 11 genes, which may be expected given the different stimuli involved (Poly IC as compared to IL-6), plus the different species and gestational time-points.

### 3.5 Acute 3 h IL-6 exposure increases microglia motility and chemokine secretion *in vitro*

**Fig. 4:**
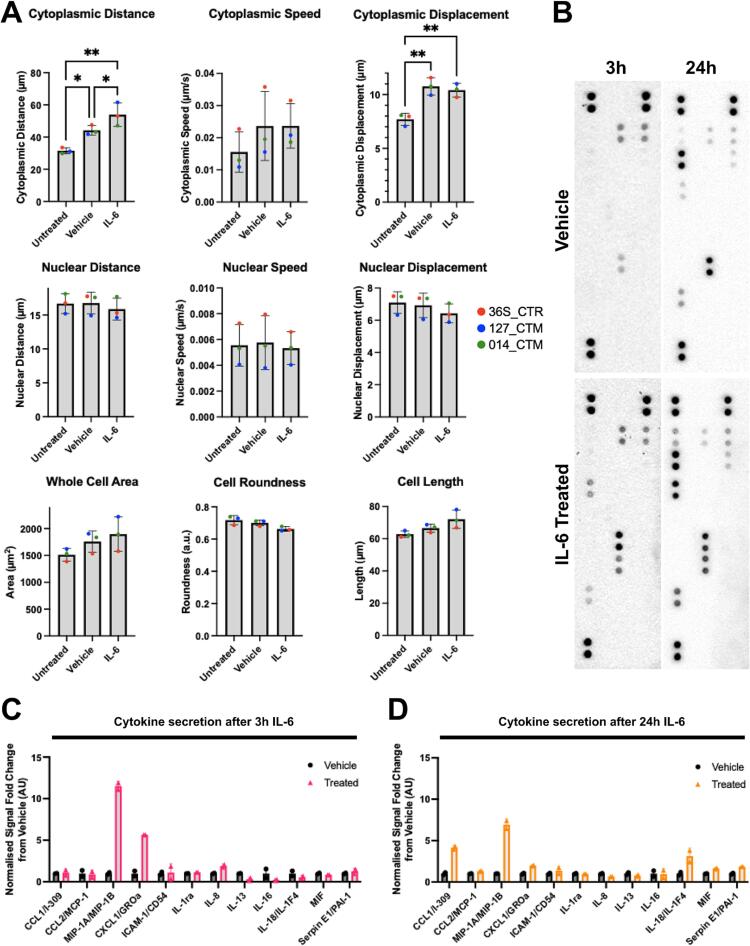
Acute IL-6 increases time-dependent changes in cytokine and chemokine secretion from human MGLs *in vitro*. MGL motility and secretome is altered in response to IL-6. (A) Metrics of MGL motility and morphology over 2 h of live imaging, having been exposed to vehicle, IL-6 100 ng/ml or untreated for 180 min. 5 % FDR Benjamini method corrections formatted as follows: *p < 0.05, **p < 0.01, ***p < 0.001, and ****p < 0.0001; not significant, not labelled. Bar graphs plotted as mean with standard deviation (SD) error bars, and points coloured by donor line: red (M3_CTR), blue (127_CTM) and green (014_CTM), all averaged from N = 3 harvest replicates. (B) Representative images of dot blot cytokine profiles secreted from vehicle and IL-6 stimulated MGLs analysed using the human cytokine array. N = 3 separate harvest media samples were pooled into one sample per condition from M3_CTR_37S. Corresponding cytokine and chemokine coordinate labels are available in Supplementary Table 22 and supplementary Fig. 5. (C-D) Signal quantification of cytokine signals from dot blots presented in B, shortlisted for detectable cytokines and chemokines and split into 3 h (C) and 24 h (D) datasets. IL-6 not shown since it was artificially spiked by treatment. Each point represents a technical replicate of each signal point in arbitrary units, which were normalised to positive control reference spots and fold change calculated from averaged vehicle value within each time point.

Both our RNAseq and qPCR data provide evidence for an increase in *IFR8* expression after IL-6 exposure in human MGLs. In mice, microglia-specific deletion of *IRF8* results in cells with fewer, shorter branches and reduced motility, consistent with the regulatory role of *IRF8* in microglia state (d’Errico et al., 2021). Furthermore, primary microglia from an *IRF8−/−* mouse model demonstrated attenuated ATP-induced chemotaxis in comparison to wild type primary mouse microglia (Masuda et al., 2014). Finally, a recent data from a mouse model of MIA provides evidence that IL-6 increases microglial motility *in vivo* (Ozaki et al., 2020). Based on these data, we acquired live cell imaging data to record the effect of 3 h exposure to IL-6 (100 ng/ml) on MGL whole cell (nuclear) motility, cytoplasmic specific (cytoplasm) motility and morphology, another known correlate of microglial function (Hanger et al., 2020). We observed that vehicle treatment was by itself sufficient to influence MGLs motility, as evidenced by an increase in cytoplasmic distance and displacement in both vehicle- and IL-6 treated cultures relative to untreated controls (Fig. 4A, statistics in Supplementary Table 21). Critically however, IL-6 increased mean cytoplasmic distance relative to vehicle-treated controls, suggestive of increased cytoplasmic ruffling (Fig. 4A, statistics in Supplementary Table 21). Nuclear motility, cell area and length were unchanged by either vehicle or IL-6 treatment (Supplementary Fig. 4). We confirmed these effects were not due to differences in cell number or the movement of cells in or out of the field of view (statistics in Supplementary Table 21). These data are consistent with increased *IRF8* expression (d’Errico et al., 2021) and our GO analysis.

Microglia release cytokines and chemokines to recruit additional immune cells to sites of pathology in the brain and these signaling molecules are often indicated to be dysregulated in SZ patients (Dawidowski et al., 2021, Morris et al., 2018, Purves-Tyson et al., 2021, Purves-Tyson et al., 2020). As several pathways related to cytokine secretion were specified during RNAseq GO analysis, we next aimed to gain an overview of cytokine secretion from IL to 6 exposed MGLs by using a proteome profiler array, as previously described (Garcia-Reitboeck et al., 2018) (Supplementary Table 22 and Supplementary Figure 5). Chemokine and cytokine release is clearly influenced by IL-6 stimulation at either 3 or 24 h after exposure to IL-6 (100 ng/ml) (Fig. 4C-D and Supplementary Table 23). Of the 36 cytokines and chemokines in the assay, 13 were above the limit of detection in culture supernatant. We excluded IL-6 since it was ectopically spiked into the media when the cells were stimulated with IL-6 (Fig. 4B). Although semi-quantitative, changes in cytokine and chemokine secretion may be represented as fold changes from vehicle. MIP-1α/β and CXCL1 were increased in supernatants from IL-6 exposed cultures at both time-points relative to vehicle-controls, although considerably less so after 24 h. This replicates findings that MIP-1α is robustly up-regulated in maternal serum of rodent MIA models (Brown et al., 2022, Osborne et al., 2019). CCL1/2, Serpin-E1, MIF and IL-18 were increased only after 24 h IL-6 stimulation with little difference observed at 3 h. IL-8 presented higher secretion 3 h after IL-6 simulation but not at 24 h. The anti-inflammatory cytokines IL-13 and IL-16 were reduced at both time-points relative to vehicle-controls. Finally, IL-1Ra, CCL2 and ICAM remained unchanged after IL-6 exposure at both time points. These alterations to the MGL secretome provide evidence that IL-6 stimulation of human MGLs leads to dynamic changes in specific inflammation-regulating chemokines and cytokines.

## 4 Discussion

We characterized the cell-specific responses of MGL and NPC to acute IL-6 exposure using hiPSC lines obtained from male, neurotypical donors (N = 3). Our data suggest two main findings; first, that hiPSC-derived MGL and NPC cells show clear differences in IL6Ra signaling capabilities and second, that exposure of MGLs to IL-6 recapitulates molecular and functional phenotypes of relevance for psychiatric disorders, particularly schizophrenia, consistent with evidence from genetic (Perry et al., 2021), blood biomarker (Allswede et al., 2020) and animal models (Smith et al., 2007) that link this cytokine with increased risk for schizophrenia.

We observed that both hiPSC-derived forebrain NPCs and mature neurons express very low levels of *IL6R*, resulting in their limited ability to respond to IL-6 treatment in monoculture, as evidenced by the absence of STAT3-phosphorylation and *IL6, JMJD3* and *TNF* expression changes post-IL-6 exposure. These data are seemingly at odds with those from hiPSC-derived mature neurons derived using a similar differentiation protocol, in which IL-6 exposure resulted in transcriptional and morphological phenotypes (Kathuria et al., 2022). One important difference between this work and our own is the age at which these cells were exposed to IL-6: specifically, D18 NPCs vs D25-27 neurons (Kathuria et al., 2022). Although we confirmed *IL6R* expression is absent in forebrain neurons at D50, it may be possible that *IL6R* mRNA is expressed transiently between days 25–50, or only protein levels are present, which is not detectable by RNAseq. Therefore, we cannot rule out that D25-27 neurons derived by this protocol can prompt an IL-6 response and that differences in the timing of IL-6 exposure may lead to differential results (Estes and McAllister, 2016). A second difference is the dose and length at which these cells were exposed to IL-6: 3 h and 24 h of 100 ng/ml IL-6 herein as compared to 48 h to 1 µg/ml IL-6 (Kathuria et al., 2022). It is important to note here that just as observed in animal MIA models, differences in the intensity and duration of immune activation will lead to variations in results. In this context we sought to find the minimum concentration of IL-6 that our cells would respond to *in vitro*, at least in monoculture, which is 100 ng/ml and the dose with which we continued to characterize the cell’s IL-6 response. We did not carry out RNAseq on our IL-6 exposed NPC RNA, so we cannot discount a non-canonical response to IL-6 that is independent of the IL6Ra-STAT3 pathway at higher concentrations of IL-6. More experiments are necessary to address these questions.

Furthermore, our data strongly suggest MGLs in co-culture with neural progenitors and additional cell types are necessary to study the influence of IL-6 on NPC-specific development when using D18 NPCs under the conditions tested in our study. In this context, we observed secretion of sIL6Ra protein from MGLs but minimal sIL6Ra secretion from NPCs at two separate time points, suggesting that in a co-culture paradigm, secretion of the sIL6Ra by MGLs may enable NPCs to respond to IL-6 via *trans*-signaling *in vitro*. In support of this view, data from a transgenic mouse model provide evidence that targeted inhibition of CNS *trans*-signaling via sIL6Ra mitigates several relevant neuropathological hallmarks previously associated with NDDs, including impaired neurogenesis, blood brain barrier leakage, vascular proliferation, astrogliosis and microgliosis (Campbell et al., 2014). These data support the view that sIL6Ra *trans*-signaling may be a relevant pathogenic mechanism of IL-6 in both glial and non-glial cell types (Campbell et al., 2014). In addition, sIL6Ra is present (irrespective of inflammatory conditions) in human CSF at 1.92–3.03 ng/ml (Azuma et al., 2000, Hans et al., 1999). This not only indicates that IL-6 *trans*-signaling is possible in the human brain but also supports the data presented in this study, that MGL sIL6Ra secretion reaches concentrations relevant to human CSF in culture media after 24 h and is independent of IL-6 stimulation. Further studies are therefore now required to confirm in our system whether forebrain NPCs can activate *trans*-IL6 signaling in the presence of sIL6Ra protein.

Our second main observation is the genes up-regulated by acute IL-6 exposure in our hiPSC-derived MGLs significantly overlap with genes increased in *post-mortem* brain issue from SZ patients, but not ASC or BD (Gandal et al., 2018). However, these genes did not overlap with risk genes for SZ, ASC and BD, as identified by GWAS. This could suggest that the relationship observed between IL-6 and these disorders may be driven by environmental factors leading to abnormal levels of IL-6 (Allswede et al., 2020, Careaga et al., 2017, Graham et al., 2018, Meyer, 2014, Mueller et al., 2021, Potter et al., 2023, Rasmussen et al., 2021, Rasmussen et al., 2019, Rudolph et al., 2018). We are unable however, to disregard the effect of genetics as a potential mediator of this relationship, as all variants and genes located within the histocompatibility complex (MHC) locus are excluded from the MAGMA analysis due to the complex linkage disequilibrium structure in this region, although many IL-6 response genes are in this locus. Ultimately, the development of new methods to translate findings from the MHC into neurobiological risk mechanisms for complex disorders could advance our understanding of the role of the MHC in psychiatric disorders.

Key up-regulated DEGs in this study, such as *HSPA1A/B*, *Rel* and *IRF8* are responsible for maintenance of microglial homeostasis, core microglial signatures and stress responses (Galatro et al., 2017, Gosselin et al., 2017, Olah et al., 2020), consistent with their differential expression after IL-6 stimulation. The observed overlap with up-regulated SZ gene sets, particularly with *HSPA1A/B* and *NFKB2* overlap genes (Supplementary Table 20), suggests a link between IL-6 exposure, microglial signature pathways and SZ pathogenesis consistent with *post-mortem* gene expression data in brain tissue from individuals with SZ (Murphy et al., 2021, Volk et al., 2019). Importantly, microglial genes are also reported to be down-regulated in both RNAseq and qPCR studies of human cortical *post-mortem* brain tissue from individuals with SZ (Gandal et al., 2018, Snijders et al., 2021). Taking an example directly relevant to our study, *IRF8* expression is down-regulated in *post-mortem* human cortical brain tissue from individuals with SZ (Gandal et al., 2018, Snijders et al., 2021). In contrast to these data, we observed an increase of *IRF8* expression after 3hr acute IL-6 exposure, accompanied by increased cytoplasmic motility and time-dependent increases in chemokines and cytokines, findings consistent with the documented role of IRF8 in enabling microglia to adopt a pro-inflammatory gene signature in disease (Ransohoff and Engelhardt, 2012). In contrast, Ormel and colleagues used PBMCs transdifferentiated to induced microglia (iM) to identify two clusters of iM cells using mass cytometry, that were enriched only in donors with a diagnosis of SZ (Ormel et al., 2020). Of these, one cluster was characterized by elevated protein levels of CD68, Cx3cr1, HLA-DR, P2RY12, TGF-β1 and importantly, IRF-8 (Ormel et al., 2020). These data illustrate the possibility of confounding factors in the interpretation of microglial gene expression changes in *post-mortem* brain tissue from individuals with schizophrenia, for example, prolonged antipsychotic exposure (Chan et al., 2011) plus the end-of-life effect after a long-term disease on tissue (Mccullumsmith et al., 2014). Further studies are therefore required to characterize how IL-6 impacts on specific microglia states of relevance to SZ via mass cytometry and/or single-cell sequencing approaches using both *post-mortem* brain tissue and hiPSC cellular models with appropriate functional assays.

Our observation that acute IL-6 stimulation is associated with up-regulation of the OXT receptor (*OXTR*) and it’s overlap in the up-regulated SZ gene set (Gandal et al., 2018) (Supplementary Table 20), links with evidence that polymorphisms in the *OXTR* gene are linked to the pathogenesis of both SZ (Broniarczyk-Czarniak et al., 2022, Nakata et al., 2021), but also ASC (de Oliveira Pereira Ribeiro et al., 2018, Francis et al., 2016). Intriguingly, studies in rodent primary microglia suggest OXT suppresses inflammatory responses following LPS stimulation *in vitro* (Inoue et al., 2019). Furthermore, in mice, treatment with an OXTR agonist reduces perinatal brain damage by specifically acting on microglia (Mairesse et al., 2019). Further studies are therefore required to investigate the role of OXTR signaling in regulating MGL responses to IL-6 in our human model system, including studies in patient-derived cell lines.

Limitations of the current study should also be noted. As mentioned above, the response of each cell type presented here lies within the context of an acute IL-6 treatment in a monoculture, in the absence of a genetic background for a relevant psychiatric or neurodevelopmental disorder. In this context, birth cohort studies report the association between *average* IL-6 exposure across gestation, hence describing the impact of a cumulative exposure to IL-6 on brain and behavior phenotypes (Graham et al., 2018). Furthermore, we have previously reported differential effects of acute IFNγ exposure on gene expression in forebrain NPCs from individuals with or without a diagnosis of SZ (Bhat et al., 2022). It will therefore be important to investigate both chronic IL-6 exposure and include patient-derived hiPSC lines in future studies to address these issues. Although, *in vitro* cultures are an artificial system, they are nonetheless a useful reductionist tool that permit the investigations of precise interactions between specific cell types, which is the rationale behind their use in this study. We acknowledge, however, the need for further studies using more complex 3D culture methods such as microglia-containing organoids to extend our observations herein (Zhang et al., 2022).

Gene expression results can vary significantly as a function of individual donors in hiPSC studies. As such, we acknowledge that the modest number of donors used in the present study (N = 3) may influence our ability to find statistically significant differences. For example, IL-10 expression in 3hr D1 MGLs is not statistically significant, but in fact has a fold change (∼5-fold) higher than 3 h D14 MGLs (∼3-fold). Therefore, although the results obtained in this study may not reach statistical significance, this does not necessarily mean an absence of biological significance, and vice versa. This is specifically important when discussing cases of receptor expression changes, such as *IL6R* and *IL6ST* expression in NPCs, which demonstrate robust fold change differences despite a lack of statistical significance (Fig. 1B: *IL6R* hiPSC vs D18 = 0.137 fold change*, IL6ST* hiPSC vs D18 = 6.50-fold change)*,* and the effects of IL-6 treatment as mentioned previously. Replication studies with larger sample sizes will be required to confirm or disprove our findings. In addition, evidence from rodent models suggests functional, structural, and transcriptional sex-specific microglial differences (Guneykaya et al., 2018, Han et al., 2021, Villa et al., 2018). Our experiments were carried out using three individual clones per donor, from a total of N = 3 male donor lines, hence we cannot discount genotype- or sex-specific IL-6 responses by the select few donors chosen here. Our sample size is however consistent with existing studies of the impact of IL-6 on neurodevelopment using hiPSC models (Kathuria et al., 2022). Combined with unique differentiation and/or cytokine exposure protocols reported by different laboratories, there is substantial risk that the reproducibility and hence, validity of mechanistic data from hiPSC models will be compromised (McNeill et al., 2020). Replication of our results by multiple groups using common hiPSC reference lines (e.g. corrected KOLF92) will therefore be an important advance for this field (Volpato and Webber, 2020).

In conclusion, hiPSC-derived MGLs can respond to IL-6 via canonical IL6Ra signaling monoculture but monoculture NPCs cannot, due to their limited *IL6R* expression. The response of MGLs to IL-6 phenocopies molecular changes of relevance for SZ, consistent with the documented associations between IL-6 levels and risk for SZ (Khandaker et al., 2014, Perry et al., 2021, Schwieler et al., 2015). Our human model data also replicates key microglia findings from animal models of MIA, with relation to the microglial transcriptome of newborn pups from a PolyI:C MIA model (Matcovitch-Natan et al., 2016), IRF8-dependent microglia motility (d’Errico et al., 2021, Masuda et al., 2014) and maternal MIP-1α serum secretion (Brown et al., 2022, Osborne et al., 2019). Collectively, our data underline the importance of studying microglial cells to understand the influence of IL-6 on human neurodevelopment and to elucidate cellular and molecular mechanisms that link early life immune activation to increased risk for psychiatric disorders with a putative neurodevelopmental origin.

## Declaration of Competing Interest

The authors declare that they have no known competing financial interests or personal relationships that could have appeared to influence the work reported in this paper.

## Data Availability

Data will be made available on request.
